# Unmasking the invaders: NLR-mal function in plant defense

**DOI:** 10.3389/fpls.2023.1307294

**Published:** 2023-11-20

**Authors:** Susanna Anbu, Velushka Swart, Noëlani van den Berg

**Affiliations:** ^1^ Department of Biochemistry, Genetics and Microbiology, University of Pretoria, Pretoria, South Africa; ^2^ Hans Merensky Chair in Avocado Research, Forestry and Agricultural Biotechnology Institute, University of Pretoria, Pretoria, South Africa

**Keywords:** NLRs, sensor NLR, helper NLR, resistosome, defense, decoy

## Abstract

Plants possess an arsenal of immune receptors to allow for numerous tiers of defense against pathogen attack. These immune receptors can be located either in the nucleocytoplasm or on the plant cell surface. *NLR* gene clusters have recently gained momentum owing to their robustness and malleability in adapting to recognize pathogens. The modular domain architecture of an NLR provides valuable clues about its arms race with pathogens. Additionally, plant NLRs have undergone functional specialization to have either one of the following roles: to sense pathogen effectors (sensor NLRs) or co-ordinate immune signaling (helper or executer NLRs). Sensor NLRs directly recognize effectors whilst helper NLRs act as signaling hubs for more than one sensor NLR to transduce the effector recognition into a successful plant immune response. Furthermore, sensor NLRs can use guard, decoy, or integrated decoy models to recognize effectors directly or indirectly. Thus, by studying a plant host’s NLR repertoire, inferences can be made about a host’s evolutionary history and defense potential which allows scientists to understand and exploit the molecular basis of resistance in a plant host. This review provides a snapshot of the structural and biochemical properties of the different classes of NLRs which allow them to perceive pathogen effectors and contextualize these findings by discussing the activation mechanisms of these NLR resistosomes during plant defense. We also summarize future directives on applications of this NLR structural biology. To our knowledge, this review is the first to collate all vast defense properties of NLRs which make them valuable candidates for study in applied plant biotechnology.

## 1 Introduction

Plants possess an arsenal of immune receptors to allow for numerous tiers of defense against pathogen attack. These immune receptors are located either in the nucleocytoplasm (Lüdke et al., 2022) or on the plant cell surface (Böhm et al., 2014). The strategic location of these receptors aid in targeting pathogen effectors which can translocate into the apoplast or the host cytoplasm. Dynamic antagonism between the plant and pathogen is best epitomized in the zig-zag model of plant defense which articulates that plants possess two lines of defense, namely pattern-triggered immunity (PTI) and effector triggered immunity (ETI) to act concurrently as a continuum to ward off pathogens (Jones and Dangl, 2006). PTI is a basal immune response induced by the recognition of pathogen-associated molecular patterns (PAMPs) by pattern recognition receptors (PRRs) (Bigeard et al., 2015). ETI is a robust immune response activated by resistance genes (*R* genes) which encode either intracellular nucleotide-binding leucine rich repeat (NLR) proteins or receptor-like/receptor kinase proteins (RLP/RKPs) which directly or indirectly recognize pathogen effectors (Cui et al., 2015). This activation of ETI is manifested as a localized cell death called the hypersensitive response (HR), which allows the plant to cordon off pathogen infection to prevent systemic spread (Dalio et al., 2021). NLRs are one of the molecular players activating immune signaling to drive this HR phenotype.

*NLR* gene clusters have recently gained momentum owing to their robustness and malleability in adapting to recognize pathogens. The modular domain architecture of an NLR provides valuable clues about its arms race with pathogens. Based on its N-terminal domain structure, NLRs can be grouped into four subgroups, namely, 1. Toll/Interleukin-1-like receptor (TIR) -NLRs (TNLs), 2. Coiled-coil (CC)-NLRs (CNLs), 3. Resistance to powdery Mildew 8 (RPW8-like) CC-NLRs (RNLs) (Qi and Innes, 2013) and more recently 4. Pepper CNL-Group 10 (G10-CC)-NLRs, termed as ancient and autonomous NLRs (ANLs) (Lee et al., 2021) of which the latter two are subdivisions of CNLs. The centrally conserved domain includes a central nucleotide-binding adaptor shared by APAF-1, R proteins, and CED-4 (NB-ARC). The C-terminal domain consists of a leucine-rich repeat region (LRR), which has been attributed to confer dual functions of NLR auto-inhibition and pathogen detection (Sukarta et al., 2016). The aforementioned canonical domains constitute a classical NLR architecture, however, a subcategory of NLRs can carry non-canonical domains due to the integration of an effector target, which are referred to as NLR-integrated domains (NLR-IDs) (Cesari et al., 2014; Kroj et al., 2016; Marchal et al., 2022a). Additionally, some NLRs possess atypical or missing domains but still retain functions in plant immunity (Meyers et al., 2002; Nandety et al., 2013).

Flor (1971) represented the interaction between an *R* gene and its corresponding effector (avirulence gene (*Avr*)) through the gene for gene model. Although Jones and Dangl (2006) have been credited for the conception of ETI, Flor’s gene for gene concept echoes a receptor-ligand model for NLR-effector interactions (Flor, 1971; Van Der Biezen and Jones, 1998). Here, NLRs function as singletons to co-ordinate sensing and immune signaling. In contrast, some plant NLRs have undergone functional specialization to have either one of the following roles: to sense pathogen effectors (sensor NLRs) or co-ordinate immune signaling (helper or executer NLRs) (Jubic et al., 2019). Helper and executor NLRs are distinguished by the number of sensor NLRs that they interact with. An NLR is termed as helper if it can recognize a wide array of sensors NLRs, with such helper NLRs constituting vast NLR networks (Adachi et al., 2019b; Ao and Li, 2022). In contrast, an executer NLR is confined to interact with only one, predefined sensor NLR partner (Jubic et al., 2019). These NLR associations form an active complex defined as a resistosome (Burdett et al., 2019). This resistosome model challenges the one-on-one interaction to rather view plant immunity as a network.

**Figure 1:**
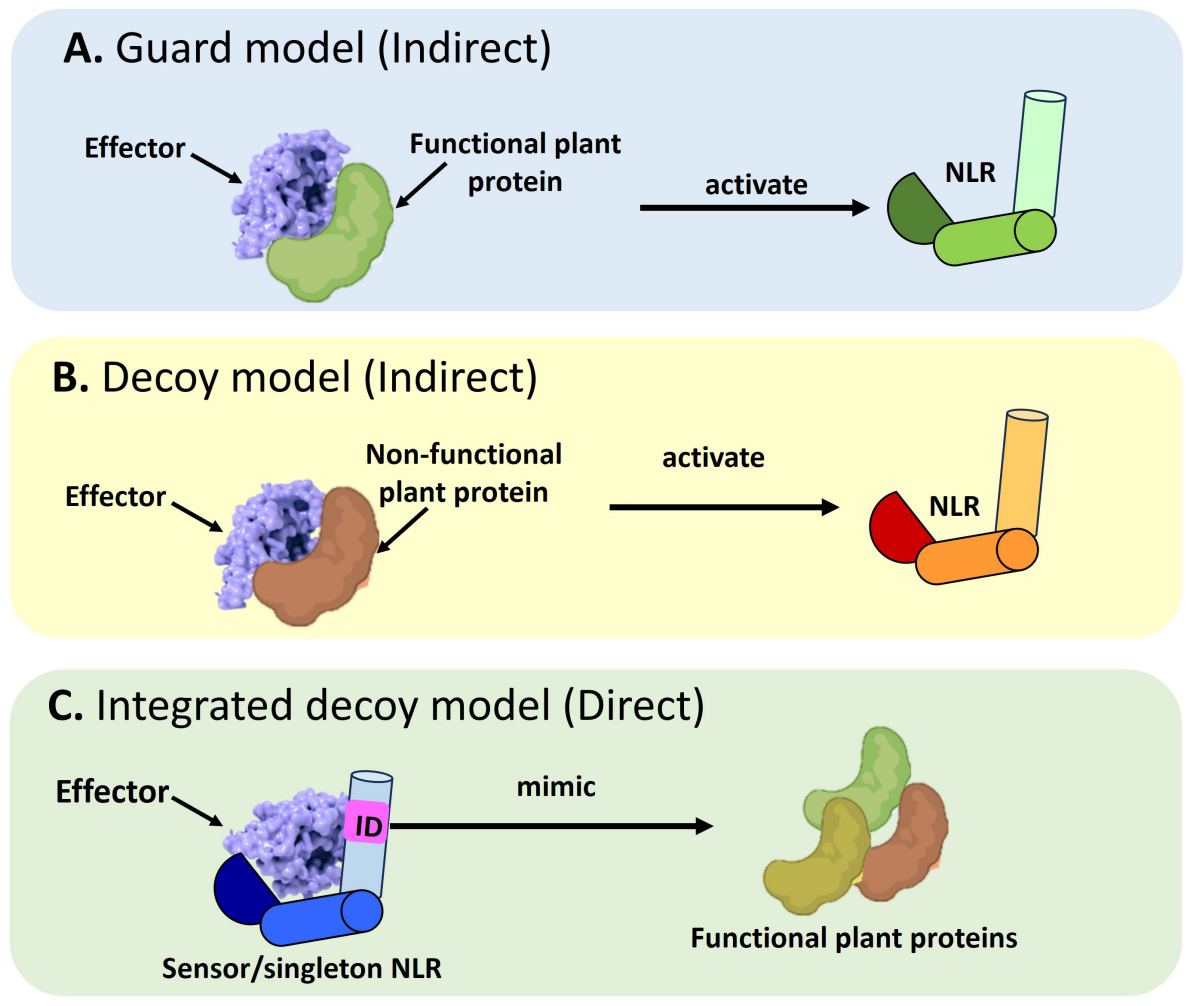
Schematic representation of the different modes of Nucleotide binding-Leucine rich Repeat NLR mediated effector recognition. NLRs can recognize pathogen effectors indirectly through **(A)** The guard model where the interaction between an effector and a functional plant protein (guardee) will trigger the activation of an NLR which will target the pathogen effector for degradation. The second mode of indirect recognition occurs via **(B)** The decoy model where a non-functional, truncated plant protein recognizes a pathogen effector to activate NLR-mediated immune signaling. Alternatively, a direct mode of effector recognition can occur through **(C)** The integrated decoy model whereby a sensor or singleton NLR incorporates an integrated domain (ID). This ID is a mimic of functional plant protein domains which serve as the originally intended targets of the pathogen effector. Such integration of the ID equips the NLR to impound pathogen effectors to divert them away from their originally intended plant target.

NLRs use the guard, decoy, or integrated decoy models to recognize effectors directly or indirectly (Cesari et al., 2014) (**Figure 1**). In the guard model, NLRs “guard” a functional plant protein (guardees), which are targeted by effectors (**Figure 1A**). This recognition between the guardee and effector will activate an NLR-mediated immune response. An *Arabidopsis* CNL called Suppressor of MKK1 MKK2 2 (SUMM2), provides a well-studied example (Zhang et al., 2012; Zhang et al., 2017c). Under healthy conditions, a mitogen-activated protein (MAP)-kinase cascade will result in the phosphorylation of Mitogen-activated Protein Kinase 4 (MAPK4) which in turn results in the phosphorylation of Calmodulin-binding Receptor-like Cytoplasmic Kinase 3 (CRCK3). However, the *Pseudomonas syringae* effector, HopAI1 can prevent the phosphorylation of MPK4 and subsequently CRCK3 (Zhang et al., 2012; Zhang et al., 2017c). The NLR protein SUMM2 senses the disruption of an immune signaling MAP kinase cascade via CRCK3 (guardee) (Zhang et al., 2017c). The decoy model operates in the same mode, except a non-functional decoy protein acts as a structural mimic of guardees to recognize an effector (**Figure 1B**). In the integrated decoy model, NLR-IDs use the same mode of operation as decoys, except the ID responsible for effector recognition is physically integrated into the NLR which facilitates a direct effector recognition (**Figure 1C**). Thus, by studying a plant host’s NLR repertoire, inferences can be made about a host’s evolutionary history and defense potential which allows scientists to understand and exploit the molecular basis of resistance in a plant host.

This review provides a snapshot of the structural and biochemical properties of the different classes of NLRs which allow them to perceive pathogen effectors and contextualize these findings by discussing the activation mechanisms of these NLR resistosomes during plant defense. We also discuss future directives on the applications of this NLR structural biology. The review is distinct from Fick et al. (2022b), who provide a summary of *NLR* regulation at the gene level.

## 2 Anatomy of an NLR

**Figure 2:**
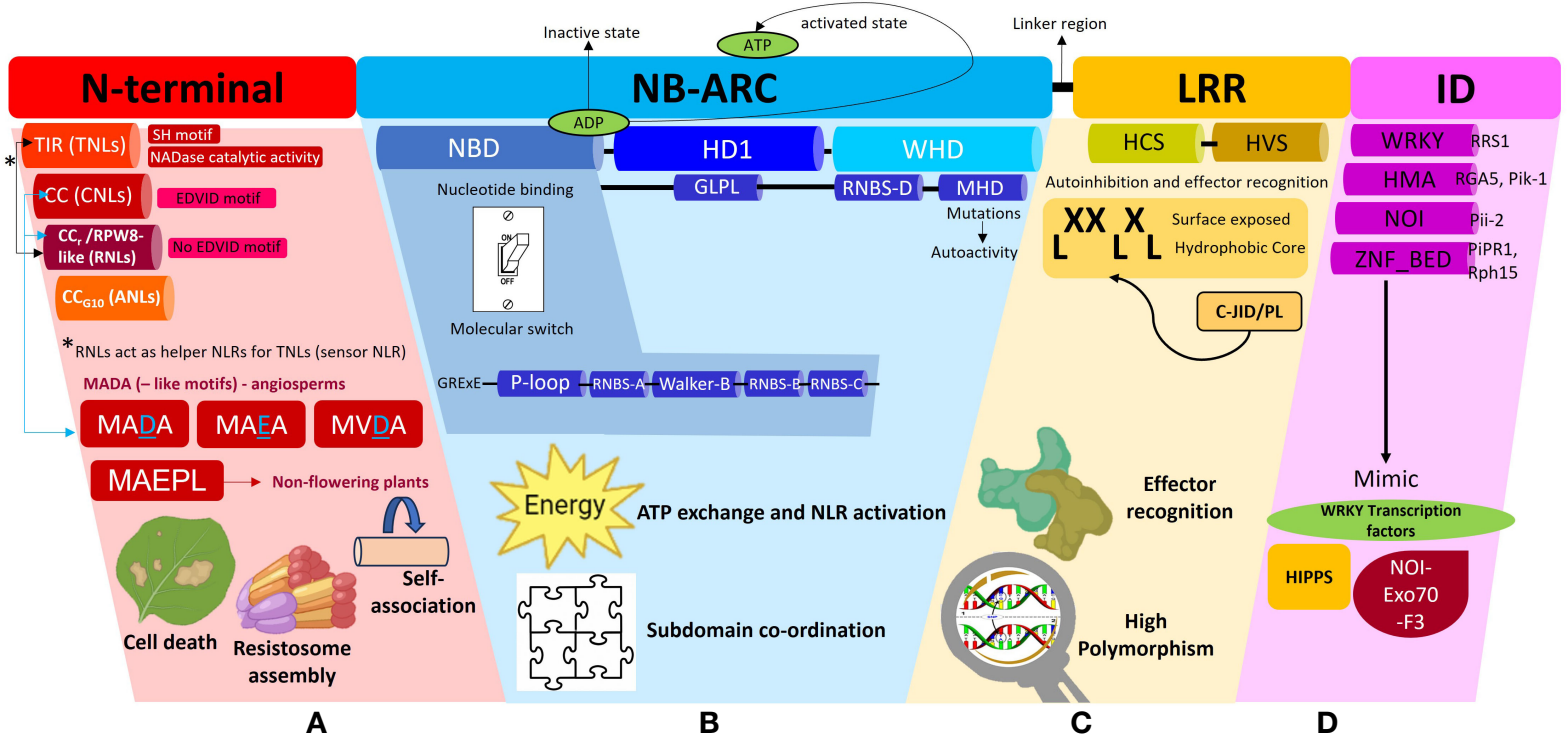
A schematic representation of the different subdomains which constitute a Nucleotide binding-Leucine rich Repeat (NLR) protein and the associated functions of each domain. This diagram is to be used in conjunction with the textual analysis presented in the Anatomy of an NLR section to better visualize the positioning of each sub-domain and associated motifs. **(A)** The N-terminal region can comprise of the Toll/Interleukin-1-like receptor (TIR), Coiled-coil (CC), Resistance to powdery Mildew (CC_r_) or Pepper CNL-Group 10 (CC_G10_) domains which dictate the classification of the NLRs into their respective subclasses, TNLs, CNLs, RNLs or ANLs. All TNLs contain the TIR domain marked by the presence of an SH motif responsible for driving cell death signaling. TNLs possess NADase catalytic activity to hydrolyze NAD^+^ to form NAD^+^ derived molecules which act as downstream signaling molecules during plant defense. CNLs possess an EDVID motif to stabilize an NLR-NLR self-association interaction during resistosome assembly whilst RNLs lack this EDVID motif. RNLs act as helper NLRs for sensor TNLs exclusively via a dedicated pathway. CNLs and RNLs possess MADA and MADA-like motifs responsible for cell death induction. MADA and MADA-like motifs are present in angiosperms whilst a derivative of this motif, MAEPL, is commonly found in non-flowering plant lineages. **(B)** The central nucleotide-binding adaptor shared by APAF-1, R proteins, and CED-4 (NB-ARC) can be subdivided into three subdomains comprising of a nucleotide binding domain (NBD), a helical domain 1 (HD1) and a winged helix domain (WHD). The NBD domain possesses a walker A motif (also known as P-loop), RNBS-A domain, Walker B motif, RNBS-B and RNBS-D domains, all of which work in tandem to facilitate ADP/ATP exchange. A short linker region connects the NB-ARC and LRR domains **(C)** The leucine repeat region (LRR) is a site of high polymorphism and consists of repetition of alternating hydrophobic (leucine) and hydrophilic amino acid residues (LxxLxLx). The LRR domain can be sub-divided into two regions, namely, a highly conserved segment (HCS) and a highly variable segment (HVS). A non-canonical jelly roll and Ig-like domain or post LRR (C-JID/PL) domain contributes to effector recognition. **(D)** Some sensor NLRs contain the integrated domain **(ID)** which mimic domains from functional plant proteins such as the WRKY transcription factors, Heavy-metal-associated isoprenylated plant proteins (HIPPs), integrated NO_3_ induced (NOI) proteins. Some examples of IDs include WRKY, heavy metal associated (HMA) domain, NOI and zinc finger (ZnF) BED domains.

NLRs possess a modular architecture encompassing a total of three domains (N-terminal, central NB-ARC and C-terminal LRR domains) (**Figure 2**) that act in tandem to function as a molecular switch. This allows the NLR to switch between the resting state in the absence of a cognate effector to an active immune signaling state in the presence of a pathogen (Takken and Goverse, 2012). Co-expression of the individual NLR domains have shown that they can be reconstituted into a functional, full-length protein (Moffett et al., 2002; Leister et al., 2005). This suggests that these NLR domains were originally present as separate proteins and acquired over time to evolve into a single multidomain immune receptor. The Rosetta Stone Hypothesis stipulates that the fusion of separate protein domains unravels a hidden interaction between these unrelated domains (Marcotte et al., 1999). In line with this principle, this fusion into a single NLR protein is likely to have conferred higher fitness costs by reducing the entropy of the reaction to make pathogen recognition more energy efficient (Staal and Dixelius, 2007; Jacob et al., 2013). Each of these domains confer the NLR with a distinct set of biochemical properties which dictates the immune signaling pathway that it will take. Thus, to better understand NLR function in plant defense, it is vital to first understand the role of each modular domain.

The N-terminal region of NLRs can comprise of either a TIR, CC, RPW8-like or CC_G10_ domain which allows categorization into their respective groups (**Figure 2A**). This region is vital in mediating downstream signal transduction after NLR activation (Takken and Goverse, 2012). Research conducted thus far has shown that TNLs are more ubiquitous in solanaceous and brassicaceous crops whilst cereal crops mainly possess CNLs (Bai et al., 2002). The CC domain within CNLs is characterized by a heptad repeat pattern responsible for forming the coiled-coil structure (Bentham et al., 2018). Within this domain, some CNLs possess a negatively charged conserved Glu-Asp-Val-Ile-Asp (EDVID) motif (Rairdan et al., 2008; Mazourek et al., 2009). Mutagenesis experiments directed at this motif in the potato immune receptor Rx1 have shown loss of protein function by hindering the intramolecular interaction with the NB-ARC and LRR domains (Rairdan et al., 2008). Another important motif characterized in the N-terminal regions of CNLs and RNLs, is a type of executioner domain comprising of a Met-Ala-Met-Asp-Ala (MADA) motif, responsible for the induction of cell death during HR (Adachi et al., 2019a). The MADA motif refers to an N-terminus consensus sequence comprising of “MADAxVSFxVxKLxxLLxxEx” amino acid residues. It is present in helper NLRs to mediate resistosome and pore formation during immunogenic cell death. Studies have shown that a short fragment containing the motif is sufficient to trigger cell death (Collier et al., 2011; Adachi et al., 2019a; Chia et al., 2022). A derivative of this motif, known as the CC_OG3_ domain comprising of a Met-Ala-Glu-Pro-Leu (MAEPL) amino acid motif, is commonly found in non-flowering plant lineages, whilst the MADA and MADA-like motifs (MVDA, MAEA) are found within angiosperms (**Figure 2A**) (Chia et al., 2022). Bentham et al. (2018) have extensively reviewed the structural and biochemical properties of CC domains, however the main takeaway points underscored that CC domains confer NLRs with any of the following functions, namely, induction of programmed cell death (PCD) (Maekawa et al., 2011), self-association (El Kasmi et al., 2017) or interaction with a co-factor (Cesari et al., 2014). The RPW8-like domain is a subclass of the CC domain, which contains a coiled-coil domain devoid of the EDVID motif (Sukarta et al., 2016) and a putative transmembrane N-terminal domain (Zhong and Cheng, 2016). RNLs are highly conserved amongst plant species and act as helper NLRs for TNLs (Collier et al., 2011). More recently, a novel cluster of CC-NLRs were found in pepper termed as ancient and autonomous NLRs (ANLs) (Lee et al., 2021). ANLs possess a coiled-coil domain which confers the NLR with auto-active functions. Analysis of the N-terminal regions demonstrates that ANLs with non-auto-active functions possess deletions within the alpha1 helix, thereby asserting the importance of this helix for cell death functions (Lee et al., 2021).

The TIR domain on the other hand has been shown to play a role in protein-protein interaction during the formation of an NLR resistosome. The crystal structures of TIR domains have been extensively reviewed by Ve et al. (2015). In terms of conservation, the Ser-His (SH) motif appears to be ubiquitous across plant TIR domains to mediate self-association or recognition of other TIR domains during resistosome formation during ETI (Burch-Smith and Dinesh-Kumar, 2007; Ve et al., 2015). Once the TIR domain mediates self-association, it confers the TIR-NLR with NADase catalytic activity. This occurs when TIRs undertake hydrolysis of NAD^+^ to form NAD^+^ derived molecules and a variant cyclic-ADP-ribose product (v-cADPR) which acts as a downstream signaling molecule in plant defense (Horsefield et al., 2019; Wan et al., 2019; Essuman et al., 2022). Additionally, SH amino acid residues within the TIR domains have shown to drive cell death signaling function in TNLs (Zhang et al., 2017a). For a detailed review on TIR domain functions in plant immunity, readers are directed to Lapin et al. (2022); Maruta et al. (2023) and Locci et al. (2023).

The central NB-ARC domain can be subdivided into three subdomains comprising of a nucleotide binding domain (NBD), a helical domain 1 (HD1) and a winged helix domain (WHD) (**Figure 2B**) (Leipe et al., 2004; Wendler et al., 2012). The NBD domain possesses a walker A motif (also known as P-loop), RNBS-A domain, Walker B motif, RNBS-B and RNBS-D domains, all of which work in tandem to facilitate the ADP/ATP exchange within the NB-ARC domain to mediate conformational changes from a resting state to an active state in response to effector recognition (van Ooijen et al., 2007; Williams et al., 2011; Bernoux et al., 2016). Mutagenesis studies at the walker A motif site which replaced the charged lysine residue with alanine or arginine residues demonstrated a complete loss of nucleotide binding (Hanson and Whiteheart, 2005). Resolved crystallography structures have demonstrated that NLRs are kept in an inactive state through the presence of an ADP residue between the HD1 and NBD grooves. This, can be disturbed upon mutations in the Met-His-Asp (MHD) motif located in the WHD, resulting in constitutive auto-activation of NLRs (Sukarta et al., 2016). The MHD motif is also essential in coordinating the interactions between the subdomains within the NB-ARC, upon effector recognition (Hanson and Whiteheart, 2005). A flexible linker region then connects the NB-ARC domain to the C-terminal LRR region. An in-depth description of how NB-ARC domains are involved in signal transduction during plant immunity activation, has been provided in a review by van Ooijen et al. (2007).

The C-terminal end of an NLR possesses the LRR domain which consists of repetition of alternating hydrophobic (leucine) and hydrophilic amino acid residues (LxxLxLx) (**Figure 2C**). The leucine motifs form a hydrophobic core, with the residues in between them exposed to the surface (Reubold et al., 2014). These surface residues account for the variability located in the LRR domains in contrast to the highly conserved N-terminal and NB-ARC domains. Thus, the LRR domain can be sub-divided into two regions, namely, a highly conserved segment (HCS) and a highly variable segment (HVS) (Liu et al., 2022). Positive selection pressures exerted on the variability of these surface residues influence the NLR’s recognition specificity. This variability confers the LRR with dual functions of auto-inhibition and pathogen effector domain recognition. Random mutagenesis of LRR regions have been shown to impact NLR recognition specificity (Lindner et al., 2020; Huang et al., 2021a). However, few effectors have been demonstrated to directly interact with the LRR domain, implying more mechanisms’ involvement in effector recognition, on the other end of the spectrum, a direct interaction with the LRR does not always guarantee NLR activation (Padmanabhan et al., 2009). In the interaction between the tomato immune receptor Swf-F and the nematode effector SPRYSEC19, it was proven that the effector’s recognition of a seven C-terminal repeat within the LRR did not activate the receptor (Postma et al., 2012). The wheat immune receptor Powdery Mildew Resistance 3 (*Pm3*) gene possesses an unusually large LRR domain which has been classified as an “island domain” which forms extending loops on the exterior of the canonical LRR domain (Koller et al., 2018). The LRR domain of the potato immune receptor Gpa2 possesses basic residues which facilitates self-recognition by binding to its own N-terminal and NB-ARC domains for auto-inhibition (Slootweg et al., 2013).

The aforementioned sections review the structural properties of a classical NLR structure. However, mutations within NLR sequences or alternative splicing processes can influence the coding of truncated NLRs, some of which have shown to retain the same defense functions as their canonical, full-length counterparts. A truncated version of the *Arabidopsis* NLR RPS4 containing a functional N-terminal TIR domain but lacking the NB-LRR domain has been demonstrated to trigger immunity (Williams et al., 2014; Saucet et al., 2015). Another truncated NLR in *Arabidopsis*, TN2 lacks an LRR domain; functional studies however proved TN2 to remain functional through the aid of helper NLRs (Wang et al., 2021b). Similarly, the Response to the bacterial type III effector protein HopBA1 (RBA1) NLR in *Arabidopsis*, contains only the TIR domain yet this was sufficient to trigger cell death in response the *Pseudomonas syringae* effector HopBA1 (Nishimura et al., 2017). These examples along with many other reports illustrate the precarious modularity of the NLR tripartite architecture, proving that NLRs have maximized their adaptive potential to still retain plant immunity functionality despite truncation (Chen et al., 2021b; Son et al., 2021; Cox, 2022).

### 2.1 IDentity theft

NLRs possess a tripartite architecture, amongst which the LRR domain has been implicated in effector recognition. In addition to this domain at the C-terminal region, some sensor NLRs possess non-canonical domains known as IDs which arise due to the integration of effector targets into NLRs (**Figure 2D**). These IDs are involved in direct and indirect effector recognition to sequester the pathogen effector to deviate it from its original plant target. Some well-known IDs include the zinc-finger BED, kinase, integrated NO_3_ induced (NOI), WRKY and heavy metal associated domain (HMA) (Kroj et al., 2016; Sarris et al., 2016; Bailey et al., 2018; Stein et al., 2018). Although the presence of IDs confers great fitness advantage to NLRs, the frequency of NLR-IDs in many NLR clades is low. Cereal crops possess three NLR-ID clades reflecting a high abundance of IDs which are designated as major integration clades (MIC) (Bailey et al., 2018). A comparison of orthologous NLR-ID clades shows that the exchange of IDs within NLRs is a continual process to garner a high diversity of IDs (Bailey et al., 2017; Bailey et al., 2018). Species within *Triticeae* possess an expanded repertoire of IDs. A phylogenetic analysis of orthologs and closely related paralogs of *Triticeae* NLRs revealed clustering with high bootstrap support demonstrating common ancestry of the shared ID (Bailey et al., 2017). This also suggests that such an ID fusion has been under selective pressure to maintain a functional fusion within the plant host. One such example of conservation was illustrated between NLR proteins from the *Triticeae* and *Brachypodium* (Bailey et al., 2017). This framework equips the plant with an assortment of novel effector recognition specificities to better defend itself against pathogen attack. This section will review some significant examples of NLR-IDs and how they have been weaponized by the plant to nullify pathogen attack.

### 2.2 WRKYing together

**Figure 3:**
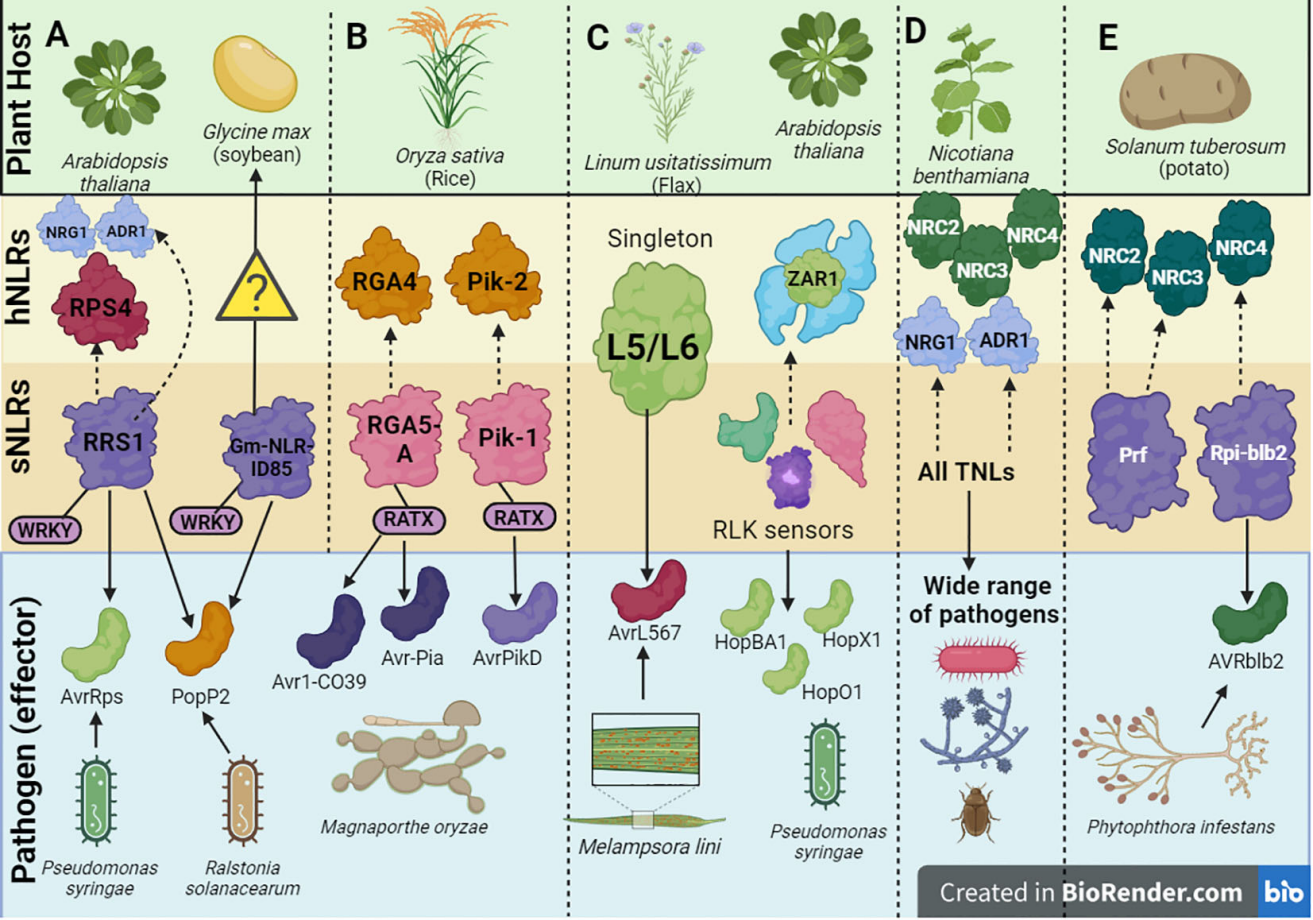
A schematic depiction of a few examples of helper-sensor Nucleotide binding-Leucine rich Repeat (NLR) pairs and networks discussed in this review, along with their corresponding pathogen effectors. **(A)** The *Arabidopsis thaliana* helper-sensor NLR pair RPS4/RRS1. In this interaction, the sensor NLR, RRS1, recognizes two bacterial effectors AvrRps and PopP2 from *Pseudomonas syringae* via its WRKY domain and transduces an immune signaling relay to the helper NLR RPS4. Upon effector recognition, RRS1 can also transduce an immune signaling relay via the NRG1/ADR1 helper NLR network. The *Glycine max* NLR, Gm-NLRID85, can also recognize the bacterial effector PopP2 via its WRKY domain. It is unclear whether Gm-NLRID85 functions as a singleton NLR or interacts with an unknown helper NLR to induce immunity. **(B)** *Oryza sativa* contains two well characterized helper-sensor NLR pairs, RGA4/RGA5 and Pik-2/Pik-1. The sensor NLRs, RGA5 and Pik-1 contain the RATX or heavy metal associated domain (HMA) to recognize *Magnaporthe oryzae* effectors. RGA5 recognizes Avr1-CO39 and Avr-Pia, whilst Pik-1 recognizes AvrPikD. RGA5 and Pik-1 transduce signals to their respective helper NLR counterparts, RGA4 and Pik-2. **(C)** Singleton NLRs from *Linum usitatissimum* called L5 and L6 recognize the AvrL567 effector from *Melampsora lini*. The singleton NLR, ZAR1, from *A*. *thaliana* recognizes a family of Hop effectors from *P. syringae*. This interaction can be strengthened by the action of receptor-like protein kinase (RLK) sensors. **(D)** In solanaceous plants like *Nicotiana benthamiana*, all TNLs act as sensor NLRs to recognize effectors from a wide range of pathogens. This effector recognition by TNLs is transduced into an immune signaling relay via one of two RNL helper NLR networks, namely the NRC network or NRG1/ADR1 network. **(E)** Prf and Rpi-blb2 are two NLRs in *Solanum tuberosum* which act as sensor NLRs and operate via the NRC network. Rpi-blb2 recognizes Avrblb2 from *Phytophthora infestans*.

A pair of *Arabidopsis* NLRs, RRS1/RPS4 have been shown to recognize the bacterial effectors AvrRps and PopP2 from *P. syringae* pv*. pisi* and *Ralstonia solanacearum*, respectively through an integrated WRKY domain in the C-terminal region of RRS1 (**Figure 3A**) (Le Roux et al., 2015; Sarris et al., 2015; Huh et al., 2017). This RRS1^WRKY^ domain is hypothesized to mimic the DNA-binding domains in plant WRKY transcription factors (TFs) involved in activation of defense genes (Sarris et al., 2015). Typically, the WRKY TFs bind to a W-box consensus sequence in the promoters of defense genes through a WRKYGQK motif to activate or repress transcription (Xu et al., 2020). The WRKY TFs have shown to enhance the plant’s response to biotic and abiotic stresses, hence it is unsurprising that targeting these TFs intensifies the pathogen’s virulence (Rushton et al., 2010).

One study has characterized the structural basis of the RRS1^WRKY^-AvrRps4 complex, revealing that the AvrRps4 effector directly interacts with the WRKYGQK motif in RRS1 (Mukhi et al., 2021). A comparison of the RRS1^WRKY^-AvrRps4 (Mukhi et al., 2021) and RRS1^WRKY^-PopP2 (Zhang et al., 2017b) structural complexes shows an overlapping β2-β3 segment within the WRKY domain binding site that could be responsible for effector binding. Thus, the integration of the WRKY domain into RRS1 allows it to sequester effectors and subsequently divert them from binding to their original targets (WRKY TFs) (Mukhi et al., 2021). However, there are some nuances in this binding interaction which can cascade to a successful or unsuccessful immune response.

The RRS1/RPS4 pair exists in an inhibited resting state until a lysine residue within the WRKY domain is acetylated, which switches it from an inhibited complex to an activated one to trigger an immune response (Le Roux et al., 2015). One study has generated a library of known IDs that can be utilized to screen with pathogen effectors to better identify virulence targets (Landry et al., 2021). Here, PopP2 was shown to physically interact with the WRKY domain within the GmNLR-ID85 in soybean. In contrast to the acetylation induced at the WRKY domain in RRS1 in *Arabidopsis* (Ma et al., 2018), the WRKY domain found in GmNLR-ID85 in soybean could not be acetylated, rendering the pathogen effector incapable of repressing GmNLR-ID85 activity. This shows that it is likely that the soybean NLR contributes to plant immunity through a different mechanism than that of RRS1.

### 2.3 An HMAzing detector!

Heavy-metal-associated isoprenylated plant proteins (HIPPs) are metal ion binding chaperone proteins which possess a characteristic isoprenylation motif and a heavy metal associated (HMA) domain (de Abreu-Neto et al., 2013). The pairing of these two distinct domains has conferred HIPPs with a unique advantage of aiding the plant to cope with increasing levels of heavy metal residues in the environment due to pesticide use and industrialization (Singh et al., 2016). Considering the importance of HIPPs in plants, it is unsurprising that they serve as targets for pathogen effectors. One study exploited a yeast-2-hybrid system to identify the plant proteins targeted by a *Magnaporthe oryzae* effector called Avr-PikD (Oikawa et al., 2020). It was demonstrated that four small heavy metal-associated domain containing (sHMA) proteins were bound by Avr-PikD, of which two are members of the HIPP family (Oikawa et al., 2020). Maidment et al. (2020) have demonstrated the structural basis for the interaction between Avr-Pik effector variants and OsHIPP19 proteins via the HMA domain.

As a countermeasure to this effector’s mode of action, NLRs have integrated the HMA domain within their structures to exploit an integrated decoy mechanism to trap effectors. In rice specifically, two NLRs, RGA5 and Pik-1 have been characterized to possess the HMA domain (Ashikawa et al., 2008; Okuyama et al., 2011). As of date, the HMA domain has been identified in four botanical families (Brassicaceae, Fabaceae, Rosaceae and Poaceae) (Sarris et al., 2016). In the RGA5/RGA4 system, RGA5 serves as the sensor whilst RGA4 serves as the helper NLR (**Figure 3B**). RGA5 possesses the HMA or related to *Arabidopsis* trithorax1 (RATX) domain at the C-terminal region located after the LRR domain. Alternative splicing of RGA5 generates two different isoforms of RGA5, namely RGA5-A which contains the HMA domain and RGA5-B which lacks the HMA domain (Cesari et al., 2013). In line with this, RGA5-A conferred resistance to *M. oryzae* isolates expressing both the Avr1-CO39 and Avr-Pia *M. oryzae* effectors.

In the Pik-1/Pik-2 system, the Pik-1 NLR possesses an HMA domain (**Figure 3B**), however this is located between the CC N-terminal region and NB-ARC domain compared to RGA5 where the domain is located after the C-terminal LRR domain (Maqbool et al., 2015). It is unclear whether the varying locations of the HMA domain within NLRs can impact the degree of effector binding affinities. Once a direct interaction occurs between the HMA domain within Pik-1 and the Avr-PikD effector, an immune signaling cascade is initiated. Studies have shown that the binding interface utilized by the effectors in both the Pik-1/Pik-2 and RGA4/RGA5 systems differ significantly (Maqbool et al., 2015; De la Concepcion et al., 2018). Effector recognition by the Pik-1/Pik-2 pair forms a tripartite complex involving the effector and the Pik NLRs rather than the negative regulation mechanisms associated with RGA4/RGA5. This could provide some biochemical basis motivating the differing HMA interfaces between the two NLR systems. It is noteworthy that the interaction between the Avr-PikD effector and the HMA domain in OsHIPP19 shows a much higher binding affinity compared to the interaction between Avr-PikD and the HMA domain in the Pik1 NLR (Maidment et al., 2020). Therefore, it is unclear at what point the mounted immune response by the integrated HMA domain can surpass the pathogenic activity of the effector.

The NLR-effector interaction associated with the WRKY and HMA domains are two of the best characterized systems to date illustrating the integrated decoy model (**Figure 1D**). Other examples of IDs include the integrated NOI, zinc-finger BED and kinase domains (Marchal et al., 2022a). The Pii-2 NLR from rice possesses an integrated NOI domain which binds to a host protein called Exo70-F3 (Fujisaki et al., 2017). Under healthy conditions, the Exo70-F3 host protein assembles into a NOI-Exo70-F3 complex which serves as the original target of the effector, Avr-Pii from *M. oryzae* (Fujisaki et al., 2017; De la Concepcion et al., 2022). Thus, by integrating the NOI domain, Pii-2 can monitor and deactivate Avr-Pii upon pathogen attack. A few studies have found that the integrated kinase motifs within NLRs demonstrate sequence similarity to kinases involved in plant immunity, whilst the integration of the zinc-finger (ZnF) BED domain into NLRs allows the capture of effectors that originally intend to target plant TFs involved in defense (Dardick et al., 2012; Kroj et al., 2016; Marchal et al., 2018; Chen et al., 2021a). The Rph15 NLR from barley and PiPR1 from rice are two examples of NLRs containing ZnF_BED IDs (Chen et al., 2021a; Liu et al., 2021b). Sarris et al. (2016) and Marchal et al. (2022a) provide an in-depth review of the ID comparisons across plant lineages.

These structural properties equip plant NLRs with the necessary molecular arsenal to carry out effector recognition and subsequent cell death in some scenarios. This recognition can be direct or indirect depending on the structural biology of the NLR, however in both instances, the NLRs are kept in a resting, inactive state in the absence of an effector. The remaining sections will delve into selected examples of direct and indirect modes of NLR-mediated effector recognition along with their associated immune signaling relay.

## 3 NLR-ending source of protection: direct recognition of effectors

During direct recognition, an effector is detected by its cognate NLR through a direct physical interaction. This is either facilitated by an NLR singleton or a sensor NLR and its co-regulated NLR partner. This helper NLR functions to transduce the sensor NLR’s effector recognition into an immune response. In some cases, this sensor NLR in this interaction possesses an ID which mimics the original plant protein target of the effector in a bid to steer the effector away (Cesari et al., 2014). Thus, direct effector recognition remains as one of the effective mechanisms to combat pathogen attack.

The interaction between the singleton flax NLR proteins L5/L6 and the variants of the flax rust effector AvrL567 is driven by polymorphism within the LRR domain, specifically the first seven and last four amino acid residues (**Figure 3C**) (Ravensdale et al., 2012). Crystal structures have ascertained that the binding interaction occurs between the two ends of the LRR domain within the curved β-sheet (Wang et al., 2007). There are amino acid contact points within this interaction which additively contribute to strengthening and stabilizing the binding interaction. Other studies have shown that the strength of the NLR-effector interaction at the amino acid level strongly correlates to the amplitude of downstream HR (Dodds et al., 2006).

Other examples of NLR singletons include, the *Arabidopsis* NLR, ZAR1, which remains one of the best characterized singleton NLRs to date. Scientists have been tentative to affirm the singleton status of ZAR1, due to the discovery of certain receptor-like protein kinase (RLK) sensors which potentially facilitate the ZAR1 mediated effector recognition of the Hop family of effectors from *P. syringae* (**Figure 3C**) (Martel et al., 2020). Thus, it is unclear if the binding interaction only involves that of ZAR1 or other RLK proteins as well. ZAR1 can exist in one of three states depending on the presence of an effector: it can exist in an inactive monomeric state in the absence of an effector, a pre-activated monomeric state upon effector recognition and a wheel-like pentameric complex to initiate cell death in response to effector recognition (Wang et al., 2019a; Wang et al., 2019b). Additionally, two TIR-NLR singleton proteins, ROQ1 from *Nicotiana benthamiana* and RPP1 from *Arabidopsis* which interact with the effectors XopQ1 and ATR1 from *P. syringae* reveal a non-canonical jelly roll and Ig-like domain or post LRR (C-JID/PL) domain which contributes to effector recognition (Ma et al., 2020). In contrast to IDs which can recognize effectors on their own, the C-JID/PL domain works with the LRR domain to recognize effectors. Ma et al. (2020) confirmed that C-JID/PL domains were exclusively found in TNLs after failure to detect the domain in non-NLR plant proteins and CNLs.

Singleton interactions are however prone to be easily overcome by the pathogen via mutations along the NLR-effector interface. Thus, NLRs functioning through the integrated decoy model also exploit a direct interaction but this mechanism is underscored by a “bait and trap” strategy which seizes the effector via an ID (Cesari et al., 2014). As of date, three sensor-helper NLR pairs, (where the sensor contains an ID) have been characterized extensively: RGA5/RGA4 (rice) (Cesari et al., 2014), Pik-1/Pik-2 (rice) (Ashikawa et al., 2008) and RRS1/RPS4 (*Arabidopsis*) (Sarris et al., 2015) (**Figure 3**).

Studies have identified two alleles of RRS1 which have differing C-terminal lengths after the WRKY domain which impact their effector recognition spectra (Ma et al., 2018; Guo et al., 2020). RRS1-R possesses a 104 amino acid extension after the WRKY domain and can perceive both AvrRps4 and PopP2 effectors whilst RRS1-S possesses an 21-amino acid long extension and can only recognize AvrRps4 (Ma et al., 2018; Guo et al., 2020). Another allelic pair RRS1B/RPS4B was shown to perceive AvrRps4 and not PopP2 (Saucet et al., 2015). A further study found that AvrRps4 bound to RRS1B^WRKY^ with a lower affinity compared to other allelic interactions (Mukhi et al., 2021). Thus, the RRS1/RPS4 pair illustrates that a careful interplay between differential interaction strengths of the effector and NLR is governed by amino acid mutations. This is suggestive that the coupling of the WRKY domain, and its extension residues mediate the direct recognition of multiple effectors.

In the interaction between RRS1/RPS4 and AvrRps4, an 88 amino acid long C-terminal region was sufficient to activate RRS1/RPS4 mediated immune signaling (Sohn et al., 2009; Sohn et al., 2012). The RPS4 executor in the RRS1/RPS4 pair possesses a C-JID/PL domain. Ma et al. (2018) have demonstrated that mutations of this domain disrupted RRS1/RPS4 triggered immunity.

### 3.1 Lending a helping hand: indirect recognition of effectors

Plant evolutionary processes to restore direct effector recognition have been slower as pathogens are able to surpass this resistance over time. Recent research has shown a wider diversity of mechanisms by which NLRs recognize pathogens indirectly which is best represented in the guard and decoy model. There are two distinctions that can be made amongst helper-sensor NLRs. Some helper-sensor NLR pairs are genetically linked in a head-to-head orientation in the genome (Narusaka et al., 2009). In contrast, “promiscuous helper NLRs” which can work in tandem with multiple sensor NLRs and vice versa, are genetically unlinked (Jubic et al., 2019).

The presence of sensor and helper NLRs as separate functional units has given rise to the concept of redundancy in plant NLR networks. This functional specialization allows sensor NLRs to undergo diversifying selection to acquire novel domains to keep up with the emergence of novel pathogen effectors during co-evolutionary cycles (Adachi and Kamoun, 2022). In contrast, helper NLRs remain conserved owing to their roles as coordinators of immune signaling and thus do not experience selection pressures. The Fluctuating Red Queen hypothesis articulates that rare host genotypes e.g., presence of certain sensor NLRs, equip the host with higher fitness compared to common genotypes as pathogens adapt to attack the most common host genotypes (Brockhurst et al., 2014). This type of negative frequency dependent selection maintains genetic variation in both the host and pathogen populations through fluctuations in allelic frequencies (Han, 2019). Thus, the presence of redundant helper NLRs coupled with specialized sensor NLRs equips the plant with resilience against pathogen effectors that may target these conserved signaling hubs.

The concept of an NLR network first arose upon the discovery of a subclade of Solanaceae helper NLRs where a group of helper CNLs called NLR-Required for Cell Death (NRC) proteins act as redundant helper NLRs to work with numerous sensor NLRs (Wu et al., 2017). This led to the conception of the first type of NLR network known as the NRC network. NRC2, NRC3 and NRC4 have been characterized in *N. benthamiana* with paralogues being characterized in other plant hosts (**Figure 3D**) (Lin et al., 2022). In the potato host, the sensor NLR Rpi-blb2 works with the helper NRC4 to illicit cell death whilst another sensor NLR Prf uses NRC2 and NRC3 to cause cell death (**Figure 3E**) (Wu et al., 2016; Wu et al., 2017).

The second type of NLR network that has been characterized is the N Requirement gene 1 and Activated Disease Resistance 1 family (NRG1/ADR1). This is an RNL type of helper NLR family required to act as helper NLRs for all TIR NLR sensors. Studies have shown a tight correlation of copy number between TIR-NLRs and RNLs like NRG1 thereby substantiating a signaling link between the two families (Liu et al., 2021a). Sensor NLRs can require either or both NRG1 and ADR1 as helpers to co-ordinate immune signaling. Some studies have illustrated an unequal genetic redundancy between NRG1 and ADR1 (Sun et al., 2021). In *Arabidopsis*, the RRS1/RPS4 pair was shown to require ADR1 for complete resistance (Saile et al., 2020). However, a comparison of an *Arabidopsis* mutant lacking *NRG1* and *ADR1* genes to a triple mutant (*adr1*, *adr1-L1*, *adr1-L2*) lacking *ADR1* genes showed the former to exhibit a more susceptible phenotype (Saile et al., 2020). This shows that *ADR1* and *NRG1* genes work in tandem rather than interchangeably to contribute to full resistance. The RRS1 sensor NLR in the RRS1/RPS4 pair was previously characterized as an NLR-ID owing to the presence of the WRKY motif (Le Roux et al., 2015; Sarris et al., 2015). It is unclear whether the RRS1/RPS4 pair working with NRG1/ADR1 produces a higher amplitude of immunity compared to the RRS1/RPS4 pair on its own.

Having established the framework of NLR networks, it is important to understand the structural biology and biochemical characteristics of helper NLRs in the NRC and ADR1/NRG1 networks that enable them to act as signaling molecules during plant defense. The first 29 amino acids within NRC4 have been shown to trigger HR during pathogen infection in potato (Adachi et al., 2019a). The same study showed these 29 amino acids to exhibit high sequence similarity to the MADA cell death causing motif in ZAR1 called MADA. Mutation experiments have validated that the loss of certain hydrophobic residues in the NRC MADA motif leads to loss of cell death activity as observed in ZAR1. However, mutating the E11 residue in NRC did not lead to a loss in cell death as witnessed in ZAR1 (Adachi et al., 2019a). It is noteworthy that prior studies on ZAR1 had characterized the E11 residue to drive Ca^2+^ channels (Wang et al., 2019a; Hu et al., 2020; Förderer et al., 2022). This shows that although NRCs may function in the same way as ZAR1 via induction of pentameric resistosome complexes to form plasma membrane pores, the biochemical basis is different.

Plants can use direct and indirect modes of effector recognition to induce the HR - which serves as the final hallmark of a successful immune response. Not all NLR-effector interactions have been demonstrated to induce an HR. This manifestation is governed by a set of fine-tuned biochemical properties and genetic processes which dictate whether the plant should invest cellular resources towards an HR.

### 3.2 Am I being too sensitive?

The sessile nature of plants has exerted selection pressures on the evolution of stringent, genetic control of plant cell death to effectively arrest pathogen proliferation. Plant cell death can be distinctly categorized into two types, namely, programmed cell death (PCD) and necrosis. It is proposed that PCD is a broad concept encompassing two classes. The first class is defined as vascular cell death whilst the second class is defined as necrotic cell death (Van Doorn et al., 2011; Midgley et al., 2022). Class one can be defined as a systematically controlled cell death vital for the survival of the plant whereas class two type necrosis is an uncontrolled, accidental plant cell death driven by necrotrophic pathogens to favor their proliferation (Gunawardena and McCabe, 2015). PCD is a cellular death that has remnants of autophagy due to the release of hydrolases from the plant vacuole which produces a “cell corpse” devoid of fluid. Necrosis on the other hand lacks autophagy and rather results in the swelling of plant organelles to result in an unprocessed, semi-fluid cell corpse (Gunawardena and McCabe, 2015). Bringing it to the context of plant defense, it is argued that HR cannot be categorized into either class owing to possessing intermediary characteristics of necrotic and vacuolar cell deaths (Coll et al., 2011). This section seeks to delve into the concept of how NLRs induce HR as a defense mechanism against pathogens.

**Figure 4:**
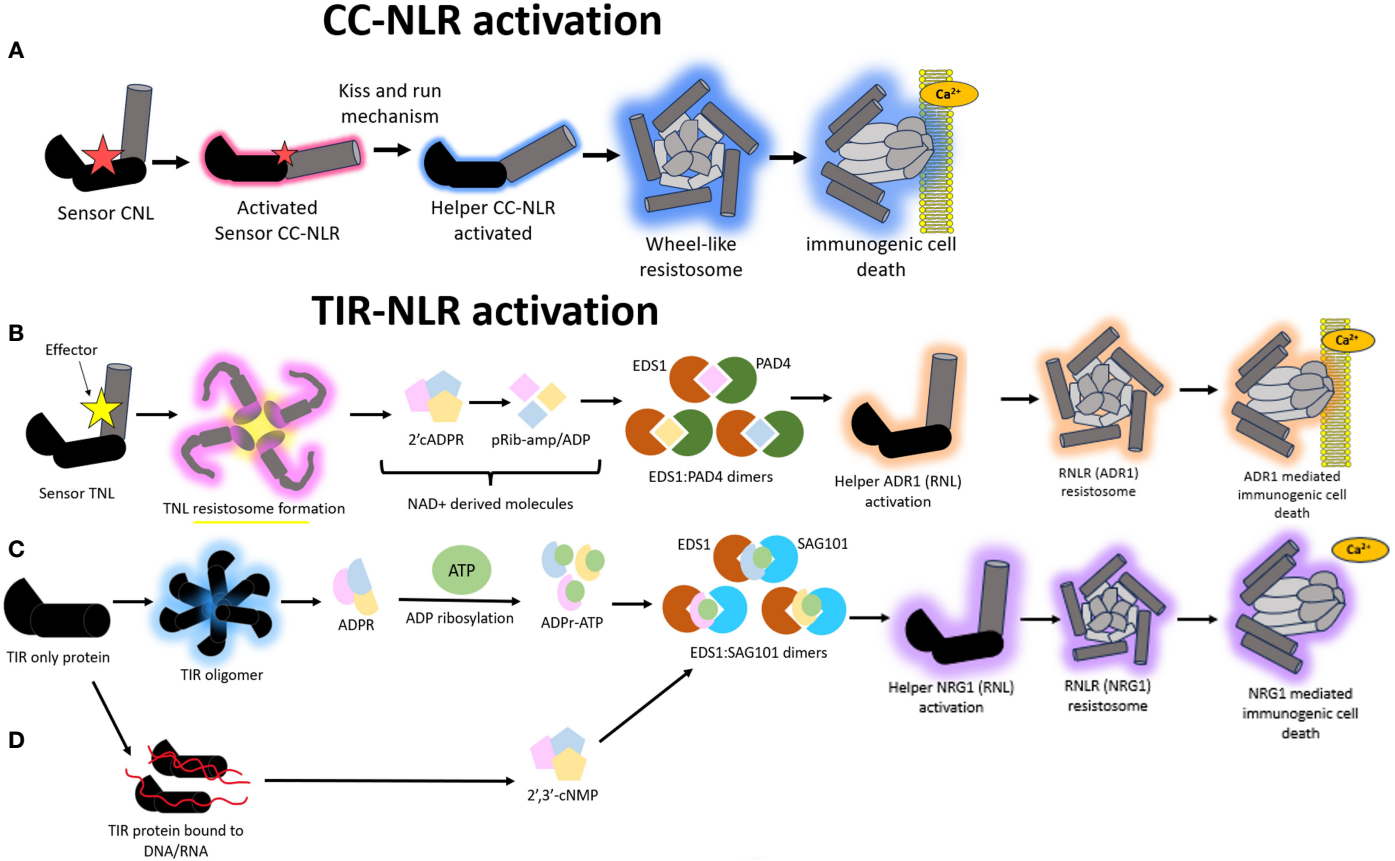
A schematic representation of the different modes of Nucleotide binding-Leucine rich Repeat (NLR) mediated hypersensitive response (HR). **(A)** Model illustrating the immune signaling relay associated with Coiled-coil nucleotide binding-leucine rich repeat (CNL) mediated activation of HR. A sensor CNL will recognize an effector and subsequently become activated. This activated sensor CNL will activate a corresponding helper CNL via a “kiss and run” mechanism which will trigger the helper CNL to self-associate and form a wheel like resistosome complex and a subsequent funnel shaped structure to pierce the plasma membrane and cause immunogenic cell death. **(B)** Model illustrating the immune signaling relay associated with Toll/Interleukin-1-like receptor nucleotide binding-leucine rich repeat (TNL) mediated activation of HR. All TNLs transduce effector recognition into an immune response using the Resistance to powdery Mildew RNL network, N Requirement gene 1 and Activated Disease Resistance 1 family (NRG1/ADR1). A sensor TNL becomes activated after recognizing an effector to form a TNL resistosome. The TNL resistosome uses its NADase activity to hydrolyze NAD^+^ to create NAD^+^ derived molecules. These molecules incorporate into a family of lipase-like proteins comprising of Enhanced Disease Susceptibility 1 (EDS1) and Phytoalexin-Deficient 4 (PAD4) to form heteromeric dimers. The heteromeric dimer complexes trigger the activation of the helper ADR1 which will trigger the formation of an ADR1 resistosome. This ADR1 resistosome will form a funnel shaped structure to pierce the plasma membrane and create a Ca2^+^ influx to illicit a HR. **(C)** In a second type of TIR mediated HR signaling, TIR domain containing proteins catalyze the formation of variant cyclic-ADP-ribose (vADPR) molecules which integrate into EDS1: Senescence Associated Gene 101 (SAG101) dimers to trigger the activation of NRG1 resistosome and subsequent funnel shaped structure which causes the formation of a Ca^2+^ channel to induce immunogenic cell death. **(D)** In a third mode of action, TIR proteins can catalyze DNA/RNA to form molecules which get integrated into the EDS1:SAG101 signaling relay to subsequently cause NRG1 resistosome formation and eventual immunogenic cell death.

CNLs activate cell death via a distinct mechanism from that of TNLs. First, sensor CNLs become activated via recognition of their cognate pathogen effectors. These activated sensor CNLs then activate helper CNLs via a “kiss and run mechanism” without incorporating themselves into the resistosome formation (**Figure 4A**) (Shepherd et al., 2023). It is unclear what biochemical basis underscores this activation mechanism. Both helper and sensor in this instance are CNLs. One notable characterization of CNL-mediated HR is ZAR1. Structural studies show that ZAR1 can form complexes with itself via homo-oligomerization to recognize effectors and co-ordinate immune signaling (Wang et al., 2019a). Crystallography structures show ZAR1 to self-associate to create a funnel-shaped pentameric resistosome to puncture a plasma membrane pore for Ca^2+^ signaling (Bi et al., 2021). The hydrophobic residues within the MADA motif in ZAR1 has been attributed to drive the pore formation whilst a negatively charged E11 residue within the motif could be driving the Ca^2+^ signaling (Jacob et al., 2021). As a result, ZAR1 has been metaphorically dubbed as the “death switch”. This “death switch” is present in a portion of helper CC-NLRs but has degenerated in sensor CC-NLRs (Adachi et al., 2019a). Thus, ZAR1 provides insight into how HR is initiated in response to NLR-mediated recognition of effectors.

There is evidence to suggest that the NRG1/ADR1 (*Arabidopsis*) network likely forms a resistosome structure mimicking that of ZAR1 to co-ordinate immune signaling. All TIR-NLRs transduce effector recognition into an immune response using the NRG1/ADR1 network, both of which are RNLs. This pathway shows remnants that are very similar to those found in animals and prokaryotes. There are two ways in which TIR-NLRs enzymatically initiate a HR upon effector recognition. In the first mode of action, a sensor TNL is activated via effector interaction to form a TNL resistosome (**Figure 4B**). This is distinct from the CNL-mediated HR where the formation of high order resistosomes is mediated by the helper CNLs as opposed to the sensor CNLs (Shepherd et al., 2023). After the TNL resistosome is formed, the TNLs hydrolyze NAD^+^ to create v-cADPR products which incorporate into a family of lipase-like proteins comprising of Enhanced Disease Susceptibility 1 (EDS1), Phytoalexin-Deficient 4 (PAD4) and Senescence Associated Gene 101 (SAG101) (Essuman et al., 2022; Lapin et al., 2022) (**Figure 4B**). These proteins can self-associate or form dimers with one another to co-ordinate Ca^2+^ signaling and elicit NRG1/ADR1 mediated HR (Wagner et al., 2013; Sun et al., 2021). There is an important distinction that happens during this signaling pathway, whereby EDS1:PAD4 dimers become the signaling components in the scenario where the v-cADPR products are derived from activity of TIR domains within NLRs. These EDS1:PAD4 dimers activate ADR1 resistosome formation and subsequent cell death (**Figure 4B**) (Maruta et al., 2023).

Alternatively, TIR proteins, which possess TIR domains but are not classified as TNLs, can also form other types of v-cADPR products of which these get incorporated into the EDS1:SAG101 signaling components to drive NRG1 resistosome formation and subsequent immunogenic cell death (**Figure 4C**). In a third and distinct mode of action, the TIR domains within TIR proteins can drive the hydrolysis of RNA/DNA for the induction of cell death, however it remains to be discerned which NLR network pathways mediate this, with the outcome resulting in the activation of the EDS1:SAG101 complex and subsequent NRG1 mediated cell death (**Figure 4D**) (Essuman et al., 2022; Lapin et al., 2022; Yu et al., 2022).

An auto-active mutant called NRG1.1 can localize to the plasma membrane for pore formation via self-associated complex whereas the wildtype version of NRG1.1 cannot undertake this (Jacob et al., 2021). ADR1s can also self-associate via the N-terminal regions to interact at the plasma membrane in a phospholipid dependent manner (Saile et al., 2021). NRG1/ADR1 do not show sequence similarity to the N-terminal MADA motif region in ZAR1, however they possess the same set of negatively charged amino acid residues (Jacob et al., 2021). These residues act as the drivers of pore formation and subsequent cell death in both NLR systems. Thus, like ZAR1, NRG1/ADR1 uses Ca^2+^ signaling to initiate the HR.

The overexpression of both RPS4 and RGA4 helper NLRs on their own trigger cell death, however overexpressing their corresponding sensor NLRs, RPS4 and RGA5 on their own did not (Cesari et al., 2014; Sarris et al., 2016). This is suggestive that the helper NLR possesses auto-activity which is repressed by the co-expression of the sensor NLR upon effector recognition. The question then arises, how does this repression of cell death contribute to a successful HR upon pathogen recognition? An interesting observation arises in the avocado-*Phytophthora cinnamomi* system where a RGA4-like protein was found in avocado, however a homologue pertaining to RGA5 was absent (Fick et al., 2022a). It remains to be discerned whether overexpression of the RGA4 protein homologue in this system triggers cell death *in planta* and potentially responds to *P. cinnamomi* Avr proteins (Fick et al., 2022a). In contrast, Pik1/Pik2 operate co-operatively to trigger cell death with neither of them displaying auto-activity upon separate overexpression. This illustrates that although all three pairs fall under the helper-sensor model, the mechanisms by which they trigger cell death are distinct.

Although cell death mediated by NLRs is an obvious indication of plant immunity activation, disease resistance without cell death is also noteworthy. The HR is a phenotypic manifestation of a successful defense response, however owing the dynamic nature of ETI, it is likely that not all successful immune interactions manifest as such.

## 4 Engineering next level resilience

The rational design of synthetic NLRs first necessitates the discovery of candidate NLRs which show broad spectrum resistance against multiple pathogens. A tomato NLR, Mi-1.2 facilitates resistance to a nematode and arthropod simultaneously (Nombela et al., 2003; Atamian et al., 2012). Similarly, the tomato receptor Cf-2 demonstrates resistance against the fungal pathogen *Cladosporium fulvum* and the root-knot nematode *Globodera rostochiensis* (Lozano-Torres et al., 2012). Using such NLR candidates as a framework, domain swapping, structure guided, random or targeted mutagenesis experiments can be conducted to create mutant NLRs which can confer resistance to phylogenetically divergent pathogens within a plant host. **Supplementary Table 1** provides an extensive summary of all studies to date which have managed to engineer a mutant NLR with new effector recognition specificity. Some of these examples will be used to discuss common principles associated with NLR engineering.

Most NLR engineering experiments revolve around plant hosts such as potato, tomato, and rice. Two studies used an array of mutations in the potato NLR Rx to identify mutations which mitigated the necrosis associated with poplar mosaic virus (PopMV) and demonstrated increased resistance to two potato virus X strains (CPTK and CPKR) (Farnham and Baulcombe, 2006; Harris et al., 2013). Owing to the infancy of the study, authors tentatively warned that the trade-offs associated with these mutations may not confer the same advantages in a natural agricultural setting. Also in the potato host, another study tested eight single residue mutations within a potato NLR, R3a, which was shown to confer recognition of a *Phytophthora infestans* effector, Avr3a while a N336Y mutation conferred R3a with the novel ability to detect an effector called PcAvr3a from *Phytophthora capsici* (Segretin et al., 2014). These mutations from R3a were subsequently transferred to the tomato ortholog, I2 to mutate the N- terminal domain to create I2^I141N^ (Giannakopoulou et al., 2015). This I2 NLR has been characterized to confer resistance in tomato against *Fusarium oxysporum* f. sp. *lycopersici* (van der Does et al., 2019). Thus, the wildtype I2 demonstrates a weak response to Avr3a, whilst the mutated version I2^I141N^, showed a stronger response against two Avr3a splice variants. These results together demonstrated that I2^I141N^ exhibited partial resistance against *P. infestans* and an expanded recognition spectrum to *F. oxysporum* f. sp. *lycopersici* effectors (Giannakopoulou et al., 2015).

Other studies have engaged in rational NLR design using guard, decoy or integrated decoy NLR systems. Some modifications include the editing of IDs or the swapping out of IDs between NLRs or swapping cleavage sites within plant proteins to modify the activation of guard or decoy systems in response to wider pathogen stimuli. A proof-of-concept study in the Pik-1/Pik-2 system in the rice-*M. oryzae* pathosystem, showed how polymorphism amongst NLRs facilitated the recognition of different effector alleles (De la Concepcion et al., 2019). In this system, *Pikm* alleles have been demonstrated to have the ability to recognize any of the Avr-Pik effector variants, owing to the presence of the HMA domain (De la Concepcion et al., 2018). In contrast, the *Pikp* allele can only recognize one of these variants, assumably due to the lack of the HMA domain which acts as the site of polymorphism. However, Cesari et al. (2022) were able to engineer new effector recognition spectra by molecularly engineering an ID into an NLR to extend its spectrum of recognition to other effectors and not just different alleles of the same effector. This study exploited two rice NLRs, Pikp-1 and RGA5 which both possess an HMA ID to recognize effectors. Pikp-1 recognizes the *M. oryzae* effector Avr-PikD whilst RGA5 recognizes the effectors Avr-Pia and Avr1-CO39. Effector binding residues from the HMA domain in Pikp-1 were introduced into the HMA domain in RGA5 to create two mutants, RGA5_HMAm1 and RGA5_HMAm2. These effector binding residues were determined from prior structural studies of HMA-effector interactions (De la Concepcion et al., 2018; Guo et al., 2018). Co-expression studies in *N. benthamiana* illustrated that RGA5 variants carrying this engineered domain recognize a new effector, Avr-PikD in addition to their original effectors.

The RPS5/PBS1 system in *Arabidopsis* operates using a guard model where the RPS5 NLR is activated upon *P. syringae* AvrPphB effector mediated cleavage of the plant protease PBS1 (Kim et al., 2016). Studies have been directed towards modifying this PBS1 cleavage site to enable cleavage by different pathogen effectors to allow the RPS5/PBS1 system to be activated upon wider pathogen stimuli (Kim et al., 2016; Helm et al., 2019; Pottinger et al., 2020). The replacement of seven amino acids within a cleavage site in PBS1 allowed activation of RPS5 in response to turnip mosaic virus (TuMV) infection (Pottinger et al., 2020). The same authors also modified a soybean ortholog of PBS1 via domain swapping to enable cleavage by a nuclear inclusion protein a (NIa) protease from soybean mosaic virus (SMV) which activated RPS5. Another study in the soybean host involved integrating a cleavage site for NIa protease from SMV into soybean PBS1 paralogues which activated an unknown soybean NLR assumed to be paralogous to RPS5 (Helm et al., 2019). Using the same principle, a tobacco etch virus (TEV) NIa protease cleavage site was engineered into PBS1 which allowed immune activation in response to TEV (Kim et al., 2016). Pottinger and Innes (2020) provide an in-depth understanding of how the RPS5/PBS1 interaction can be manipulated for translational applications.

Several studies have sought to conduct natural variation analysis to identify NLR variants possessing mutations which confer higher fitness to the host via effector recognition (Zhu et al., 2017; De la Concepcion et al., 2018). One study demonstrated that four polymorphic sites within the LRR domain of tomato NLR Sw-5b can confer broad spectrum resistance against a suite of American origin tospoviruses via recognition of a conserved 21 amino acid domain within a viral movement protein (NSm) (Zhu et al., 2017). Asian and European origin strains of the tospovirus however did not elicit an HR-type cell death, suggesting that these strains likely adopt a novel mechanism to surpass Sw-5b mediated defense.

Mutations within *NLR* alleles dictate protein conformations which favor activation. A study conducted in flax NLRs demonstrated the structural basis of two receptors, L6 and a weaker counterpart, L7 (Bernoux et al., 2016). Using site directed mutagenesis regions within the TIR and NB domains were found to contain polymorphic residues responsible for the weaker activity of L7 in contrast to L6 in both effector independent and dependent scenarios (Bernoux et al., 2016). This consensus aided in the conception of an “equilibrium-based switch model” where NLRs engage in a dynamic cycle between an inactive (ADP bound) state to active (ATP bound) state in the absence of an effector, rather than a consistent inactive state. This allows the NLR to be in a state that is poised to switch to the activation state upon pathogen entry. Thus, the rational design of NLRs can also be motivated by mutations governing signaling cascades and NLR confirmations which favor quick activation.

NLRs are activated via the release of the auto-inhibition state. Therefore, by inducing mutations that promote this release, NLRs with expanded recognition spectra can be engineered (Marchal et al., 2022b). In the case of the NLR, Rx from potato, the mutagenesis of the LRR domain formed a “trigger happy” NLR which was activated in response to a wider array of pathogen signals (Farnham and Baulcombe, 2006; Harris et al., 2013). Two of these Rx mutants showed effective immunity against the notorious resistance breaking strains of potato virus X (PVX) and PopMV (Farnham & Baulcombe, 2006; Harris et al., 2013). Similarly, the tomato NLR Sw-5b was engineered to show increased resistance towards tomato spotted wilt virus (TSWV) (Huang et al., 2021a). Here, the authors, built on previous work (Zhu et al., 2017) to introduce two mutations within Sw-5b, one within the LRR and another at the N-terminal region. The coupling of these two mutations conferred the Sw-5b mutant with increased resistance against resistance breaking isolates of TSWV (Zhu et al., 2017; Huang et al., 2021a).

Other avenues of research have looked to conduct cross kingdom studies of TIR domain functions to improve TNLs’ functions against plant pathogens. Under normal conditions, an insect-transmitted phytoplasma effector known as SAP05 targets a family of GATA zinc finger transcription proteins (Huang et al., 2021b). One study has fused a GATA SAP05-dependent degron domain to the C-terminal region of the TIR-NLR, RRS1, to create a mutant RRS1-R (Wang et al., 2021a). This domain introduction has influenced the RRS1-R NLR to act as a bait to trap the SAP05 effector.

Despite research being directed towards the rational design of NLR receptors, some caveats still exist. Although the engineered rice NLR mutants RGA5_HMAm1 and RGA5_HMAm2 show extended recognition spectra in model plant species, this extended resistance against *M. oryzae* could not be observed when introduced into rice (Cesari et al., 2022). Both mutants retained their wildtype function of recognizing their original effectors, Avr-Pia, and Avr1-CO39, however they were unable to recognize any other effectors. This is an indication that high binding affinity between the HMA domain and effector alone does not trigger immune responses, suggesting that more molecular interactions beyond the HMA domain are required to stabilize the RGA4/RGA5 complex. This underscores the importance of spatial and steric positioning of effector-NLR complexes which go beyond amino-acid interfaces. It has been illustrated that the TIR-NLRs, ROQ1 and RPP1 rely on multiple effector recognition sites for a successful direct binding interaction (Ma et al., 2020; Martin et al., 2020), hence the same could be hypothesized for RGA5.

Although the past few years have culminated vital proof-of-concept research pertaining to engineered NLR receptors, some challenges remain to be tackled. A few studies have indicated that the induction of mutations or domain swapping can cause auto-immune phenotypes when transiently expressed in heterologous model systems (Białas et al., 2021; Wang et al., 2021a). The creation of new binding affinities is an important first step, however NLR-effector binding interactions are not the sole determinants of a successful response. These engineered NLRs could be functional in a niche-specific controlled system, thereby compromising its broad scale applicability. The creation of adaptive plant immune systems is one avenue that is being explored to implement broad scale applicability.

## 5 NLR we there yet?

This review has coalesced vital research pertaining to NLR structural biology to contextualize the larger picture of how NLRs operate in immune signaling pathways. This has opened the Pandora’s box of research to exploit these properties to harness NLR defense potential into a universal defense model applicable to broader systems. Research has been directed towards retooling NLR pathways to create an adaptive immune system mimicking that of higher mammals. Two main reasons have been postulated to drive research towards the creation of made-to-order NLRs. Firstly, the presence of *NLR* genes is not ubiquitous across all food cultivars. Papaya, watermelon, and cucumber are a few crops possessing a low number of NLRs (Lin et al., 2013; Li et al., 2016). This has implications for the high level of disease severity experienced by these crops. Secondly, receptor mutagenesis and domain shuffling have been the primary ways to retool the plant’s immune system (Segretin et al., 2014; Giannakopoulou et al., 2015). This poses limitations as it targets a specific pathogen isolate and as a result can be surpassed with the advent of new virulent pathogen races or strains. These reasons necessitate the need for an approach possessing greater adaptability to a wider range of pathogens. As a result, the replacement of IDs within NLRs with nanobodies has recently gained traction to build an adaptive plant immune system of defined specificity (Kourelis et al., 2023).

The impetus for Kourelis et al. (2023)’s study stemmed from creating a universal ID to generate made-to-order NLR receptors in response to a wide range of pathogen molecules. In animal adaptive immune systems, antibodies are generated in response to an exposed antigen. The study used minimal antigen-binding fragment of single-domain heavy-chain antibodies (VHHs or nanobodies) of camelids owing to their solubility and tendency to fold in correct orientations to maximize biotechnological applications (Hamers-Casterman et al., 1993; Greenberg et al., 1995; Muyldermans, 2013). Thus, the HMA domain in Pik-1 sensors was swapped out for nanobodies which were modified to bind to green fluorescent protein (GFP) or mCherry (Kirchhofer et al., 2010; Fridy et al., 2014). The successful binding interaction between this nanobody engineered Pik-1 sensor and GFP or mCherry demonstrated that this model can potentially be extended to pathogen effectors in place of the reporter tags.

Another study was able to restore NLR activity previously nullified by a pathogen effector by introducing core mutations which would allow it to surpass deactivation by the effector (Contreras et al., 2023). An effector called SPRYSEC15 binds to NRC2 to inhibit its activity, but not NRC4. Contreras et al. (2023) studied and mapped the structural basis of NRC4’s resistance to effector inhibition and introduced corresponding mutations into NRC2, which allowed the NRC2 mutant to resist inhibition by SPRYSEC15. This study was a pioneering effort in embarking on resurrection of pathogen nullified NLRs. Marchal et al. (2022b) provide an in-depth review on the emerging principles governing made-to-order NLR receptors.

It is highly likely that more studies will pave the way to generate plant antibodies using NLRs as a framework or work to integrate the structural basis of NLR-effector recognition to restabilize NLRs nullified by pathogen effectors. Embarking on such NLR engineering studies should be underscored by analyzing promising NLR candidates in stable transgenic lines to better understand their durability and to test the potential of deleterious phenotypes that could arise from overexpressing NLRs. Although the engineering of NLRs provide promise in supplying bespoke, broad-spectrum resistance in plants, the issue of durability and transmissibility needs to be addressed. Such novel immune receptors should be cautiously deployed into crops to ensure that they are not nullified by the adaptive potential of plant pathogens.

## Author contributions

SA: Conceptualization, Writing – original draft, Writing – review & editing, Investigation. VS: Supervision, Writing – review & editing, Conceptualization. NB: Funding acquisition, Supervision, Writing – review & editing, Conceptualization.
