# Supplementary material for: Unmasking the invaders: NLR-mal function in plant defense
Source: Front Plant Sci. 2023 Nov 20;14:1307294. doi: 10.3389/fpls.2023.1307294 (PMC10698375; doi:10.3389/fpls.2023.1307294)
Supplement: Supplementary file 1 [file DataSheet_1.pdf]

Supplementary Material

Unmasking the Invaders: NLR-mal Function in Plant Defence

Susanna Anbu, Velushka Swart, Noëlani van den Berg*

*** Correspondence: Noëlani van den Berg: Noelani.vdberg@fabi.up.ac.za**

1 Supplementary Table

1.1 Table 1: A list of NLR engineering studies and the associated modifications that have been conducted to confer novel effector recognition ability.

**NLR immune receptor/ Plant protein**<br><br>**(Plant Host)**	**New effector(s) recognized (Pathogen)**	**NLR Modification**	**Reference**
**NRC2^{D317K} (*Nicotiana benthamiana*)**	SPRYSEC15 (*Globodera rostochiensis*)	SPRYSEC15 binds to NRC2 to inhibit its activity, but not NRC4. The structural basis of NRC4's resistance to effector inhibition was mapped and corresponding mutations were introduced into NRC2, which allowed the NRC2 mutant to resist inhibition by SPRYSEC15.	(Contreras et al., 2023)
**Pikm-1 (*Oryza sativa*) nanobody fusion**	Viral coat protein (Potato virus X) tagged with fluorescent proteins	NLRs were used as scaffolds to form nanobody fusions capable of binding to fluorescent proteins. This allows the activation of immune responses to mediate resistance against effectors and plant virus coat proteins expressing fluorescent proteins.	(Kourelis et al., 2023)

**Pik-1 mutants (*O. sativa*)**	Avr-PikC and Avr-PikF (*Magnaporthe oryzae)*	Using knowledge of the binding interaction between Avr-Pik and osHIPP19, new Pik-1 variants were generated which could recognize Avr-PikC/F.	(Maidment et al., 2023)
**StPBS1^{Nla} (*Solanum tuberosum*)**	PVY Nla-Pro protease (Potato virus Y)	The cleavage sequence of AvrPphB within StPBS1 was replaced with a PVY Nla-pro protease domain to create StPBS1^{Nla}. This conferred the protein with stronger immunity against Potato virus Y (PVY) infection compared to StPBS1 transgenic lines. (Decoy engineering)	(Bai et al., 2022)
**Sr35 (*Triticum aestivum*)**	AvrSr35 variants (*Puccinia graminis* f. sp. *tritici*)	Random mutagenesis experiments identified a repertoire of substitutions in AvrSr35 which could potentially escape Sr35 recognition. Likewise, a set of substitutions were identified within Sr35 which could recapture AvrSr35.	(Förderer et al., 2022)
**RGA5m1 (*O. sativa*)**	AvrPikD (*M. oryzae)*	The Avr-PikD binding residues located in Pikp-1_HMA were introduced into RGA5_HMA.	(Cesari et al., 2022)
**Sr33 (*T. aestivum*)**	AvrSr50 (*P. graminis* f. sp. *tritici*)	12 amino acids in Sr50 NLR which mediated recognition of AvrSr50 was mapped and transferred to Sr33 which resulted in an AvrSr50-dependent cell death initiation in transient expression assays. (Domain swapping)	(Tamborski et al., 2022)
**Sw-5b$^{L33P/K319E/R927A}$ and Sw-5b$^{L33P/K319E/R927Q}$ (*Solanum lycopersicum*)**	Variants of Tomato spotted wilt virus (TSWV)	Owing to the two-step recognition process employed by Sw-5b, two mutations were introduced in a stepwise manner. First, a set of mutations were introduced at the R927 residue in the LRR, after which random mutagenesis was directed at the domain. The mutants	(Huang et al., 2021)

		demonstrated immunity against resistance breaking isolates of TSWV.	
**RGA5^{HMA2} (*O. sativa*)**	AvrS- and AvrPib (*M. oryzae*)	HMA domain modified to confer new effector recognition specificities to AvrS- and AvrPib	(Liu et al., 2021)
**Pm2a (*T. aestivum*)**	AvrPm2 - BgtE-5846 (*Blumeria graminis* f. sp. *tritici*)	Introduction of natural, non-synonymous SNPs and structure guided mutagenesis unraveled the variant specific nature of Pm2-mediated hypersensitive response (HR). This allowed the swapping of the AvrPm2 head epitope to the non-HR-triggering AvrPm2 family member BgtE-5846 led to gain of a HR by Pm2a.	(Manser et al., 2021)
**RRS1-R (*Arabidopsis thaliana*)**	SAP05 (phytoplasmas)	A pathogen dependent degron domain was attached to RRS1-R allowing it to become degraded by a phytoplasma effector SAP05. This resulted in depression of RRS1-R ^{slh119} auto-activity, resulting in SAP05-mediated resistance.	(Wang et al., 2021)
**PBS1 mutants (*A. thaliana*)**	Nuclear inclusion protein a (NIa) protease (from turnip and soybean mosaic viruses)	Replacement of seven amino acids within a cleavage site in PBS1 allowed activation of RPS5 in response to turnip mosaic virus (TuMV) infection. Thus, TuMV resistance was engineered into the PBS1/RPS5 decoy system. Authors also modified a soybean ortholog of PBS1 to enable cleavage by NIa protease from soybean mosaic virus (SMV), facilitating resistance against SMV. (Domain swapping)	(Pottinger et al., 2020)
**PM3A variants (*T. aestivum*)**	AvrPM3A2/F2 (*B. graminis* f.sp. *tritici*)	Site-directed mutagenesis and domain swapping.	(Lindner et al., 2020)

**PBS1 protein kinase (*Glycine max*)**	NIa (SMV)	The guard model involving the NLR RPS5 and protein kinase PBS1 in *Arabidopsis* was exploited in soybean. New cleavage sites for NIa protease from SMV was integrated into PBS1 paralogues in soybean which activated an unknown soybean NLR. (Decoy engineering)	(Helm et al., 2019)
**Pikp variants (*O. sativa*)**	Avr-Pik (*M. oryzae*)	A structure guided engineering approach was used to expand Pikp's recognition spectra to recognize variants of Avr-Pik.	(De la Concepcion et al., 2019)
**Sw-5b (*S. lycopersicum*)**	viral movement protein (Nsm) (tospoviruses)	Natural variants of Sw-5b revealed a set of four polymorphic sites within the LRR domain which conferred resistance against tospoviruses. (Natural variation analysis)	(Zhu et al., 2017)
**PBS1 kinase (*A. thaliana*)**	TEV Nla protease (Tobacco etch virus)	Replacement of the AvrPphB cleavage site within PBS1 with a Tobacco etch virus (TEV) Nla protease cleavage site, mediated the activation of HR via TEV protease.	(Kim et al., 2016)
**$I2^{I141N}$ (*S. lycopersicum*)**	Avr3a (*Phytophthora infestans)* + some effectors from *Fusarium oxysporum* f. sp. *lycopersici.*	Mutations from the potato immune receptor, R3a were transferred to the tomato orthologue I2 to create a mutated N-terminal domain in I2: $I2^{I141N}$. (targeted mutagenesis)	(Giannakopoulou et al., 2015)
**Pm3 narrow spectrum (*T. aestivum*)**	No pathogen proteins per se but elicited an HR in transient expression system.	Two amino acid substitutions in the ARC2 domain were identified in broad spectrum resistance Pm3. These substitutions were integrated into narrow spectrum Pm3	(Stirnweis et al., 2014)

		which enhanced their ability to induce an HR response in *N. benthamiana*.	
**R3a (*S. tuberosum*)**	$Avr3a^{EM}$ (*P. infestans)* and PcAvr3a4 (*Phytophthora capsici*)	Eight single mutations extended R3a response to the $Avr3a^{EM}$ isoform. N336Y mutation in NB-ARC domain conferred recognition to PcAvr3a4. (Gain of function random mutagenesis)	(Segretin et al., 2014)
**RxM1 (*S. tuberosum*)**	Additional PVX strains (CP_{TK} and CP_{KR})<br><br>PopMV coat protein (poplar mosaic virus)	Random mutagenesis was conducted to identify four mutations which affect the NB-ARC domain structure (RxS1-4M1) to recognize potato virus X (PVX) variants and mitigate poplar mosaic virus (PopMV) necrosis.	(Harris et al., 2013)
**L5 and L6 (*Linum usitatissimum*)**	AvrL567 (*Melampsora lini*)	Site directed mutagenesis of AvrL567 and domain swapping between L5 and L6 gave consensus on which specific residues mitigate or escape effector recognition.	(Ravensdale et al., 2012)
**Chimeric Pm3 (*T. aestivum*)**	pathotypes of *B. graminis* f.sp. *tritici*	A chimeric *Pm3* gene was constructed via intragenic allele pyramiding of *Pm3d* and *Pm3e* which demonstrated a broader recognition spectrum compared to the parental alleles. (Domain swapping and site directed mutagenesis)	(Brunner et al., 2010)
**Rx (*S. tuberosum*)**	Additional PVX strains and PopMV	RxM2 showed the strongest response to CP-PoMV, RxM1 showed the strongest response to CP_{KR} PVX strain (Random mutagenesis)	(Farnham and Baulcombe, 2006)

References:

BAI, R., LI, H., DU, W., NIU, N., LI, W., GAO, Q., YAO, C., ZHOU, Z., BAO, W. & CHE, M. 2022. Decoy engineering of the receptor-like cytoplasmic kinase StPBS1 to defend against virus infection in potato. *Molecular Plant Pathology,* 23**,** 901-908.

BRUNNER, S., HURNI, S., STRECKEISEN, P., MAYR, G., ALBRECHT, M., YAHIAOUI, N. & KELLER, B. 2010. Intragenic allele pyramiding combines different specificities of wheat *Pm3* resistance alleles. *The Plant Journal,* 64**,** 433-445.

CESARI, S., XI, Y., DECLERCK, N., CHALVON, V., MAMMRI, L., PUGNIÈRE, M., HENRIQUET, C., DE GUILLEN, K., CHOCHOIS, V. & PADILLA, A. 2022. New recognition specificity in a plant immune receptor by molecular engineering of its integrated domain. *Nature Communications,* 13**,** 1524-1534

CONTRERAS, M. P., PAI, H., SELVARAJ, M., TOGHANI, A., LAWSON, D. M., TUMTAS, Y., DUGGAN, C., YUEN, E. L. H., STEVENSON, C. E. & HARANT, A. 2023. Resurrection of plant disease resistance proteins via helper NLR bioengineering. *Science Advances,* 9**,** 1-9

DE LA CONCEPCION, J. C., FRANCESCHETTI, M., MACLEAN, D., TERAUCHI, R., KAMOUN, S. & BANFIELD, M. J. 2019. Protein engineering expands the effector recognition profile of a rice NLR immune receptor. *Elife,* 8**,** 1-20.

FARNHAM, G. & BAULCOMBE, D. C. 2006. Artificial evolution extends the spectrum of viruses that are targeted by a disease-resistance gene from potato. *Proceedings of the National Academy of Sciences,* 103**,** 18828-18833.

FÖRDERER, A., LI, E., LAWSON, A. W., DENG, Y.-N., SUN, Y., LOGEMANN, E., ZHANG, X., WEN, J., HAN, Z. & CHANG, J. 2022. A wheat resistosome defines common principles of immune receptor channels. *Nature,* 610**,** 532-539.

GIANNAKOPOULOU, A., STEELE, J. F., SEGRETIN, M. E., BOZKURT, T. O., ZHOU, J., ROBATZEK, S., BANFIELD, M. J., PAIS, M. & KAMOUN, S. 2015. Tomato I2 immune receptor can be engineered to confer partial resistance to the oomycete *Phytophthora infestans* in addition to the fungus *Fusarium oxysporum*. *Molecular Plant-Microbe Interactions,* 28**,** 1316-1329.

HARRIS, C. J., SLOOTWEG, E. J., GOVERSE, A. & BAULCOMBE, D. C. 2013. Stepwise artificial evolution of a plant disease resistance gene. *Proceedings of the National Academy of Sciences,* 110**,** 21189-21194.

HELM, M., QI, M., SARKAR, S., YU, H., WHITHAM, S. A. & INNES, R. W. 2019. Engineering a decoy substrate in soybean to enable recognition of the soybean mosaic virus NIa protease. *Molecular Plant-Microbe Interactions,* 32**,** 760-769.

HUANG, H., HUANG, S., LI, J., WANG, H., ZHAO, Y., FENG, M., DAI, J., WANG, T., ZHU, M. & TAO, X. 2021. Stepwise artificial evolution of an Sw-5b immune receptor extends its resistance spectrum against resistance-breaking isolates of Tomato spotted wilt virus. *Plant Biotechnology Journal,* 19**,** 2164-2176.

KIM, S. H., QI, D., ASHFIELD, T., HELM, M. & INNES, R. W. 2016. Using decoys to expand the recognition specificity of a plant disease resistance protein. *Science,* 351**,** 684-687.

KOURELIS, J., MARCHAL, C., POSBEYIKIAN, A., HARANT, A. & KAMOUN, S. 2023. NLR immune receptor–nanobody fusions confer plant disease resistance. *Science,* 379**,** 934-939.

LINDNER, S., KELLER, B., SINGH, S. P., HASENKAMP, Z., JUNG, E., MÜLLER, M. C., BOURRAS, S. & KELLER, B. 2020. Single residues in the LRR domain of the wheat PM3A immune receptor can control the strength and the spectrum of the immune response. *The Plant Journal,* 104**,** 200-214.

LIU, Y., ZHANG, X., YUAN, G., WANG, D., ZHENG, Y., MA, M., GUO, L., BHADAURIA, V., PENG, Y.-L. & LIU, J. 2021. A designer rice NLR immune receptor confers resistance to the rice blast fungus carrying noncorresponding avirulence effectors. *Proceedings of the National Academy of Sciences,* 118**,** 1-9.

MAIDMENT, J. H., SHIMIZU, M., BENTHAM, A. R., VERA, S., FRANCESCHETTI, M., LONGYA, A., STEVENSON, C. E., DE LA CONCEPCION, J. C., BIAŁAS, A. & KAMOUN, S. 2023. Effector target-guided engineering of an integrated domain expands the disease resistance profile of a rice NLR immune receptor. *Elife,* 12**,** 1-20

MANSER, B., KOLLER, T., PRAZ, C. R., ROULIN, A. C., ZBINDEN, H., ARORA, S., STEUERNAGEL, B., WULFF, B. B., KELLER, B. & SÁNCHEZ-MARTÍN, J. 2021. Identification of specificity-defining amino acids of the wheat immune receptor Pm2 and powdery mildew effector AvrPm2. *The Plant Journal,* 106**,** 993-1007.

POTTINGER, S. E., BAK, A., MARGETS, A., HELM, M., TANG, L., CASTEEL, C. & INNES, R. W. 2020. Optimizing the PBS1 decoy system to confer resistance to potyvirus infection in *Arabidopsis* and soybean. *Molecular Plant-Microbe Interactions,* 33**,** 932-944.

RAVENSDALE, M., BERNOUX, M., VE, T., KOBE, B., THRALL, P. H., ELLIS, J. G. & DODDS, P. N. 2012. Intramolecular interaction influences binding of the Flax L5 and L6 resistance proteins to their AvrL567 ligands. *PLoS Pathogens,* 8**,** 1-15.

SEGRETIN, M. E., PAIS, M., FRANCESCHETTI, M., CHAPARRO-GARCIA, A., BOS, J. I., BANFIELD, M. J. & KAMOUN, S. 2014. Single amino acid mutations in the potato immune receptor R3a expand response to *Phytophthora* effectors. *Molecular Plant-Microbe Interactions,* 27**,** 624-637.

STIRNWEIS, D., MILANI, S. D., JORDAN, T., KELLER, B. & BRUNNER, S. 2014. Substitutions of two amino acids in the nucleotide-binding site domain of a resistance protein enhance the hypersensitive response and enlarge the PM3F resistance spectrum in wheat. *Molecular Plant-Microbe Interactions,* 27**,** 265-276.

TAMBORSKI, J., SEONG, K., LIU, F., STASKAWICZ, B. & KRASILEVA, K. V. 2022. Engineering of Sr33 and Sr50 plant immune receptors to alter recognition specificity and autoactivity. *bioRxiv***,** 1-38

WANG, S., HUANG, W., DUXBURY, Z., HOGENHOUT, S. A. & JONES, J. D. 2021. Novel effector recognition capacity engineered into a paired NLR complex. *bioRxiv*, 1-29.

ZHU, M., JIANG, L., BAI, B., ZHAO, W., CHEN, X., LI, J., LIU, Y., CHEN, Z., WANG, B. & WANG, C. 2017. The intracellular immune receptor Sw-5b confers broad-spectrum resistance to tospoviruses through recognition of a conserved 21-amino acid viral effector epitope. *The Plant Cell,* 29**,** 2214-2232.
